# Loneliness in schizophrenia: Construct clarification, measurement, and clinical relevance

**DOI:** 10.1371/journal.pone.0194021

**Published:** 2018-03-22

**Authors:** Graham M. L. Eglit, Barton W. Palmer, A’verria S. Martin, Xin Tu, Dilip V. Jeste

**Affiliations:** 1 Department of Psychiatry, University of California, San Diego, La Jolla, California, United States of America; 2 Sam and Rose Stein Institute for Research on Aging, University of California, San Diego, La Jolla, California, United States of America; 3 Veterans Affairs San Diego Healthcare System, San Diego, California, United States of America; 4 Department of Neuroscience, University of California, San Diego, La Jolla, California, United States of America; Department of Psychiatry and Neuropsychology, Maastricht University Medical Center, NETHERLANDS

## Abstract

Loneliness is a highly prevalent experience in schizophrenia. Theoretical models developed in the general population propose that loneliness is tantamount to a feeling of being unsafe, is accompanied by enhanced environmental threat perception, and leads to poor physical, emotional, and cognitive functioning. Previous research has reported that loneliness is associated with poorer physical and emotional health in schizophrenia; however, few studies have directly compared loneliness and its correlates in persons with schizophrenia and non-psychiatric comparison subjects. The purpose of the current study was to evaluate similarities and differences in the construct of loneliness, the equivalency of the measurement of this construct, and similarities and differences in the pattern of external correlates of loneliness between schizophrenia and non-psychiatric comparison groups. The third version of the University of California, Los Angeles Loneliness Scale (UCLA-3) was administered to 116 individuals with schizophrenia or schizoaffective disorder and 106 non-psychiatric comparison subjects. Additional clinical and positive psychological measures were collected, as well as demographic characteristics of the two groups. Multiple groups confirmatory factor analysis revealed that the UCLA-3 was best characterized by a bifactor model in which all items loaded on a general loneliness dimension as well as one of two orthogonal method factors reflecting item wording in both groups. Furthermore, the UCLA-3 exhibited invariant measurement of these latent constructs across groups. Mean levels of loneliness were nearly a standard deviation higher in the schizophrenia group. Nonetheless, the overall pattern and strength of correlates were largely similar across groups, with loneliness being positively associated with depression, anxiety, and perceived stress, and negatively correlated with mental well-being, happiness, and resilience. Subtle differences in correlates of age, optimism, and satisfaction with life were found. Overall, loneliness appears to be distinct from other schizophrenia-related deficits and operates similarly across schizophrenia and NC groups, suggesting that theoretical models of loneliness developed in the general population may generalize to schizophrenia.

## Introduction

Loneliness is increasingly being recognized as an important contributor to health and wellness. Surveys have highlighted a near doubling of the prevalence of loneliness from 20% to 35% among U.S. adults over the past decade [1–3]. Loneliness has been identified as a major risk factor for a range of negative health outcomes, including heart disease, depression, anxiety, and Alzheimer’s disease [4]. Furthermore, recent research suggests that loneliness, among other behavior and mood changes, may represent one of the earliest symptoms of neurocognitive disorders associated with aging [5–7]. In recognition of the negative impact and growing prevalence of loneliness, the former US Surgeon General Murthy has advocated treating this “loneliness epidemic” as a major public health concern, whose impact is on par with that of cigarette smoking and obesity [2].

Individuals with serious mental illnesses, especially those with psychotic disorders, may be especially prone to loneliness. In particular, individuals with schizophrenia are subject to stigma [8] and have greater clinical (e.g., positive symptoms, negative symptoms, etc.) challenges. In addition, on average, individuals with schizophrenia experience greater socio-environmental (e.g., poverty, low rates of employment, low rates of marriage) difficulties [9, 10], and are objectively less integrated within their communities [11, 12], although their perceived sense of belonging within the community may not differ from individuals without schizophrenia living in the same community [13]. Recent surveys indicate that self-reported annual rates of loneliness among individuals with schizophrenia and other psychotic disorders (76 to 80%) are approximately 2.3 times higher than those in the general population (35%) [14, 15]. Highlighting its clinical importance, loneliness has been reported as a significant contributor to worse quality of life in schizophrenia [16, 17] and individuals with psychotic disorders cite loneliness as one of the most important challenges in their life, second only to financial concerns [18].

Conceptually, loneliness has been defined as a distressing feeling arising from the perception that one’s social needs are not currently being met [4]. Implicit in this definition is the recognition that loneliness is related to the subjective perception of social isolation and not necessarily with an objective lack of social support. Empirical research within the general population suggests that loneliness is characterized by several negative cognitive beliefs or processes, including enhanced vigilance for social threat, expectations of generally negative social interactions, and a memory bias favoring negative over positive social information. As a result, lonely individuals often experience feelings of hostility, stress, pessimism, anxiety, and low self-esteem [19, 20]. This elevated perception of environmental threat is believed to lead to chronic activation of the autonomic nervous system and the hypothalamic-pituitary adrenocortical (HPA) axis, resulting in poor physical health outcomes, particularly as regards cardiovascular health and immune functioning [4].

Prior studies suggest that while individuals with schizophrenia experience more severe levels of loneliness, their experience of loneliness may be associated with similar cognitive biases and downstream effects on emotional and physical health as found in the general population [21–25]. Specifically, loneliness in schizophrenia is associated with negative interpersonal expectations and attributions of others [26, 27], internalized stigma [22], lower self-efficacy for community life [24], lower self-esteem [24], paranoia [26, 28], depression [25], anxiety [28], hypertension [25] and abnormal hemoglobin A1c levels [25].

Overall, the existing studies suggest some similarities of the effects of loneliness among people with schizophrenia and those from the general population. However, a more fundamental question still rests unanswered regarding the nature of the basic construct of “loneliness” in schizophrenia. To our knowledge there have been no published studies of the structure of the construct of loneliness in this group of people. A related concern is whether scales for quantifying loneliness function equivalently among individuals with schizophrenia compared to people in the general population or whether loneliness measures are subject to bias across these groups. Ensuring the generalizability of the construct of loneliness and the equivalency of its measurement is a prerequisite for making cross-group comparisons [29]. Moreover, evidence suggesting that the construct of loneliness differs across groups may call into question the generalizability of models of loneliness derived from the general population to schizophrenia.

This oversight is especially problematic as there is some ambiguity over the nature of the construct of loneliness. Factor analytic studies of the most commonly administered measure of loneliness, the UCLA Loneliness Scale [30], have reported mixed findings [31], with studies reporting that one [32], versus two [33], versus three [34, 35] factors underlie this scale in non-psychiatric populations. One-factor models describe this scale as consisting of a single general loneliness dimension; two-factor models describe this scale as consisting of dimensions of “Intimate Other” (lack of romantic or particularly close relationships) and “Social Other” (lack of a group or network of friends); and three-factor models describe this scale in terms of dimensions of “Isolation” (feelings of aloneness, rejection, and withdrawal), “Relational Connectedness” (feelings of familiarity, closeness and support), and “Collective Connectedness” (feelings of group identification and cohesion). These distinct conceptualizations of the loneliness construct have implications not only for assessment of loneliness, but also its treatment. If there are multiple dimensions of loneliness, interventions may need to be tailored to address specific aspects of loneliness in different groups.

Alternatively, Russell [30] proposed a bifactor model of the third version of the UCLA Loneliness Scale, the UCLA-3, in recognition of the potential impact of variable item wording on this scale. Bifactor models allow items to load on both a common general factor and an additional orthogonal group/nuisance factor. General factors reflect the target latent construct of the scale while group/nuisance factors typically reflect content domains within the target construct or method effects related to item wording [36]. In Russell’s model, items were specified to load on a general bipolar loneliness factor, as well as one of two orthogonal method factors reflecting the direction of item wording (positively vs. negatively worded items). Russell found that this model exhibited a strong model fit across four different samples of students, nurses, teachers, and older adults (see also [37]). This suggests that, after controlling for method effects related to item wording, the UCLA-3 consists of a single general loneliness dimension.

The goals of present study were to evaluate the nature and degree of similarity of the construct of loneliness across individuals with schizophrenia and non-psychiatric comparison subjects (NCs). Specifically, the study had four key aims: (1) to determine the factor structure of one of the most widely used and well-validated loneliness scales for the general population, the UCLA-3, by evaluating the comparative fit of one, two, three, and bifactor models of this scale among NCs and individuals with schizophrenia separately; (2) to evaluate the generalizability of the latent structure of loneliness and the equivalency of its measurement across these groups; (3) to determine differences in levels of loneliness across groups; and (4) to examine the degree to which loneliness scores correlate similarly across groups with important demographic, clinical, and positive psychological variables. We hypothesized that the UCLA-3 would be best characterized by Russell’s [30] bifactor model, that this model would generalize across samples, that individuals with schizophrenia would report higher mean levels of loneliness than NCs, and that loneliness would correlate with a similar pattern of variables across the two groups.

## Methods

### Participants

The present report was based on a secondary analysis of data from an ongoing study of schizophrenia at the University of California San Diego, supported by a grant from the National Institute of Mental Health. Some of these participants’ data have been used in prior reports (for example, [38, 39]), but this report represents the first examination of loneliness within this sample. For this report, the sample was restricted to individuals with complete UCLA-3 data. The study sample consisted of 116 non-institutionalized/outpatient adults with schizophrenia or schizoaffective disorder and 106 NCs who were administered the UCLA-3. Exclusion criteria were 1) other DSM IV-TR Axis 1 diagnosis; 2) alcohol or other substance use disorder (with the exception of tobacco) within the 3-months preceding enrollment; or 3) dementia, intellectual disability disorder, or other major neurological disorder potentially affecting cognition. In addition, participants were excluded if they had a medical disability that interfered with their ability to participate in the study assessments. Diagnoses were established using the Structured Clinical Interview for the DSM-IV-TR (SCI) [40] for the schizophrenia group and using the Mini-International Neuropsychiatric Interview [41] for the NC group, administered by a trained Research Associate (RA). Participants with schizophrenia were recruited from local outpatient clinics, medical centers, private physicians and board-and-care facilities and NC participants were recruited through flyers in the community, advertisements in local media and word of mouth. Participant recruitment was balanced by age, so as to have equal numbers of participants across groups at each age binned by half decade. The study protocol was reviewed and approved by the University of California, San Diego Human Research Protections Program (Project # 101631). The above-named institutional review board specifically approved this study. All participants provided written informed consent.

### Measures and procedures

#### Sociodemographic and clinical characteristics

Age, education, gender, marital status, living status, personal and family income, ethnicity, age of onset of schizophrenia and antipsychotic type and dose (expressed in terms of the World Health Organization (WHO) Defined Daily Dose (DDD) [42, 43] were determined via interview and/or record review. Individuals who reported living alone in an apartment or house, who were homeless, or who lived in a single room occupancy were coded as residing alone, individuals who reported living with someone in an apartment or house were coded as residing with someone else, and individuals who lived in a Board and Care facility were coded as living in a Board and Care facility. Personal and family income were coded according to the following income levels: (1) < $10,000; (2) $10,000–$19,999; (3) $20,000–$34,999; (4) $35,000–$49,999; (5) $50,000–$74,999; (6) $75,000–$99,999; (7) $100,000–$149,000; and (8) ≥ $150,000. In addition, information on social status was collected via the Hollingshead Index of Social Position (HISP) [44]. The HISP is a self-report measure of social position based on occupational and educational attainment, which is combined to classify an individual as (1) upper, (2) upper-middle, (3) middle, (4) middle-lower, and (5) lower social status position. Social position is characterized for current social position, longest held social position, and highest attained social position.

#### Loneliness

All participants completed the UCLA-3 [30], a 20-item self-report measure. Each item is preceded by the stem “How often do you feel…?”. Response categories correspond to the frequency of the item and consist of “Never,” “Sometimes”, “Often,” and “Always.” Eleven items on the scale are negatively worded, with higher frequency ratings denoting greater levels of loneliness (e.g., “How often do you feel that you lack companionship?”) and nine items are positively worded, in which higher frequency ratings correspond to lower levels of loneliness (e.g., “How often do you feel part of a group of friends?”). The UCLA-3 has demonstrated strong test-retest reliability [30], internal consistency [30, 45, 46], discriminant validity [46], and convergent validity [30, 46] among non-psychiatric samples, as well as strong internal consistency among individuals with schizophrenia [26, 47].

#### Severity of psychopathology

Severity of psychopathology was measured with the Scales for the Assessment of Positive and Negative Symptoms (SAPS and SANS, respectively) [48, 49], which were administered and scored by a trained RA. In addition, all participants completed several self-report measures, including the Brief Symptom Inventory—Anxiety Subscale [50] and the Calgary Depression Scale [51]. Assessment of cognition targeted executive functions, as this domain may be particularly relevant to schizophrenia and/or its impact on everyday functioning [52–54]. The following subtests from the Delis-Kaplan Executive Functioning System (D-KEFS) [55] were used to calculate an executive functioning composite: Trail Making (Letter-Number Sequencing Task), Color Word Inhibition (Switching condition), and the Letter Fluency task (total F, A, and S trials). The raw scores within each subtest were converted to z-scores using the Normalized Rank function of SPSS 24, and coded such that higher z-scores on each subtest represented better performance; a composite mean z-score was then calculated as the average across all three subtests for each individual.

#### Positive psychological factors

Levels of positive psychological factors were measured with several self-report scales, including the four-item Happiness factor from the Center for Epidemiological Studies—Depression Scale [56], the Life Orientation Test—Revised (a measure of optimism) [57], the Connor-Davidson Resilience Scale– 10 item version [58, 59], the Perceived Stress Scale [60], and the Satisfaction with Life Scale [61].

### Data analyses

NC versus schizophrenia group differences in sociodemographic and clinical characteristics, as well as positive psychological factors, were compared using independent t-tests for continuous variables, the Mann-Whitney U test for ordinal variables, and Pearson Chi-square for categorical variables. Significance was defined as p < .05; two tailed.

Primary data analyses of the UCLA-3 involved three steps: (1) comparison of factor analytic models, (2) evaluation of measurement invariance, and (3) examination of correlates of loneliness (UCLA-3) scores within each group, and comparison of the magnitude of those correlations between groups.

Initial primary data analyses compared factor analytic models of the UCLA-3 separately among the NCs and schizophrenia groups using confirmatory factor analysis (CFA). Four factor analytic models were specified and compared using CFA: (1) a unidimensional model with all 20 items loading onto a single latent loneliness factor and was specified following Hartshorne [32]; (2) a two-factor model corresponding to that of Wilson et al. [33], with eight items loading on a “Social Other” factor and 12 items on an “Intimate Other” factor; (3) a three-factor model paralleling that of Hawkley et al. [34], in which five items were specified to load on a “Relational Connectedness” factor, 11 items on an “Isolation” factor, and four items on a “Collective Connectedness” factor; and (4) a bifactor model that corresponded to that of Russell [30] in which all items were specified to load on a general factor reflecting individual differences on the target loneliness dimension and one of two orthogonal method factors corresponding to positive and negative item wording. Table 1 presents a mapping of items in the one, two, three, and bifactor models under investigation. After establishing best fitting factor models, internal consistency of CFA-derived UCLA scales was evaluated with Cronbach’s alpha.

**Table 1:** Item loading specification in proposed factor models of the UCLA Loneliness Scale.

UCLA Item: How often do you feel…	One Factor (Hartshorne, 1993)	Two Factor (Wilson et al., 1992)	Three Factor (Hawkley et al., 2005)	BiFactor (Russell, 1996)
1. In tune with others around me	L	S	CC	L + P
2. That you lack companionship	L	I	I	L + N
3. That there is no one to turn to	L	I	I	L + N
4. Alone	L	I	I	L + N
5. Part of a group of friends	L	S	CC	L + P
6. That you have a lot in common with people around you	L	S	CC	L + P
7. That you are not close to anyone	L	I	I	L + N
8. That your interests are not shared by those around you	L	I	I	L + N
9. Like an outgoing person	L	S	CC	L + P
10. There are people you feel close to	L	S	RC	L + P
11. Left out	L	I	I	L + N
12. That your social relationships are not meaningful	L	I	I	L + N
13. No one really knows you	L	I	I	L + N
14. Isolated from others	L	I	I	L + N
15. I can find companionship when I want it	L	S	RC	L + P
16. There are people who really understand you	L	I	RC	L + P
17. Feel shy	L	I	I	L + N
18. That people are around you but not with you	L	I	I	L + N
19. There are people I can talk to	L	S	RC	L + P
20. There are people I can turn to	L	S	RC	L + P

*Note*. L = Loneliness; S = Social Other; I = Intimate Other; CC = Collective Connectedness; I = Isolation; RC = Relational Connectedness; P = Positive Wording; N = Negative Wording

Following the recommendations of Brown [29] and Kline [62], multiple goodness-of-fit indices were evaluated to determine model fit and comparative superiority. Fit indices evaluated were the model chi-square, Root Mean Square Error of Approximation (RMSEA) [63], Bentler Comparative Fit Index (CFI) [64], the Tucker-Lewis Index (TLI) [65], and the Standardized Root Mean Square Residual (SRMR). Values indicating acceptable fit on each index were as follows: RMSEA close to .06 or below; SRMR close to .08 or below; CFI close to .95 or greater; and TLI close to .95 or greater [66].

The second data analytic step involved evaluating measurement invariance of the UCLA-3 across NC and schizophrenia groups using multiple groups CFA [67]. Invariance testing in multiple groups CFA is a multi-step procedure in which a hierarchical sequence of increasingly restrictive models is evaluated. Restriction of models is achieved by imposing additional equivalency constraints on parameter estimates across groups. Models evaluated to establish measurement invariance were, in order of evaluation, a configural invariant model (with equivalent factor structure), a weak invariant model (with equivalent item-factor loadings), and a strong invariant model (with equivalent item-intercepts). Strict invariance, in which item residuals are constrained to equivalence, was not evaluated, as item residuals are comprised in part of random error variance and are thus not expected to be equivalent across groups. Moreover, Little [68] has argued that enforcing strict invariance can distort subsequent parameter estimates and most authors contend that strict invariance is not required to establish measurement invariance [29, 68, 69]. Following the establishment of measurement invariance, differences in latent construct variances and means were evaluated across groups by constraining equivalence on these latent parameters. To anticipate what will be discussed in the Results section below, mean levels of underlying latent factors were found to be different across groups. To describe mean differences in the latent loneliness construct, effect sizes of latent mean differences were calculated in terms of Hancock’s [70] latent mean difference analogue of Cohen’s d.

Criteria for measurement invariance followed the recommendations of Little [68], in which distinct criteria were used to evaluate configural invariance, weak and strong invariance, and equivalency of latent construct variances and means. Configural invariance was assessed by determining whether the same factor model with the same pattern of item-factor loadings exhibited acceptable fit across groups separately and when both groups were combined into a single, larger group. Weak and strong invariance was determined by evaluating the change in CFI values across successive models, with a change of < .01 in CFI indicating invariance across groups [71]. Finally, group differences on latent variances and means were evaluated by inspecting change in X^2^ values over successive models, with significant X^2^ change values indicating group differences in latent parameters.

Rhemtulla, Brosseau-Liard, & Savalie [72] have argued that items on Likert-type scales consisting of less than five response categories should be treated as categorical, instead of continuous, variables. As such, we used robust weighted least squares, a method appropriate for categorical variables, to estimate all CFA models [29]. Effects coding was used to set latent variable scales in all CFA models, with one exception. Owing to difficulties establishing model convergence, user-provided start values were required in the latent mean invariance multiple group CFA model, thereby necessitating standardized latent variance scaling.

The third data analytic step involved analysis of sociodemographic, clinical, and positive psychological correlates of loneliness. Pearson’s product moment correlation coefficient was calculated between the UCLA-3 and continuous variables, Spearman’s rank-order correlation coefficient between the UCLA-3 and ordinal variables, and point-biserial correlation coefficients between the UCLA-3 and binary variables. In addition, analysis of variance was conducted to explore differences on the UCLA-3 across levels of non-binary nominal variables (i.e., living situation). Comparisons between groups on the magnitude of Pearson’s product moment correlation coefficients were then conducted using Wilcox-Muska’s bootstrapped test for independent correlations [73, 74]. The Wilcox-Muska test uses a percentile bootstrap method to construct a 95% confidence interval of the difference in two independent correlations. Confidence intervals that do not overlap with 0 are interpreted as statistically significant. For Spearman’s rank-order correlation coefficients, bootstrapped 95% confidence intervals were computed separately for relevant variables within schizophrenia and NC groups. Non-overlapping 95% confidence intervals across schizophrenia and NC groups were deemed significant.

All analyses were conducted in R version 3.3.3 [75]. The lavaan package [76] was used for confirmatory factor modeling. The psych package [77] was used to compute Chronbach’s alpha on CFA-derived UCLA-3 scales and to calculate bootstrapped 95% confidence intervals around Spearman’s rank-order correlation coefficients, and the stats package [75] was used to calculate correlations with external variables. Finally, the WRS2 package [78] was used to calculate the Wilcox-Muska’s test of difference between independent correlation coefficients. See S1 File for data used in this study.

## Results

### Group differences on sociodemographic, clinical, and positive psychological variables

The NC group had significantly higher mean education higher socioeconomic status and higher personal and family income and were more likely to be married and living with someone else. NC and schizophrenia groups did not differ in age, ethnicity, or gender (Table 2). The schizophrenia group had, as expected, worse clinical and positive psychological functioning, as evidenced by higher levels of depression, anxiety, and perceived stress, and lower levels of executive functioning, mental well-being, physical well-being, happiness, optimism, resilience, and satisfaction with life.

**Table 2:** Demographic, clinical, and positive psychological characteristics of the study sample.

Characteristic or outcome	Possible range	Mean (SD), or proportion		Test
		Schizophrenia (n = 116)	Non-Psychiatric (n = 106)	
**Sociodemographic and clinical characteristics**				
Age	NA	50.77 (10.44)	51.49 (11.40)	t(220) = 0.49
Age of Illness Onset	NA	22.78 (8.26)	NA	NA
Gender (% Male)	NA	53.45%	44.34%	X^2^ (1, N = 222) = 1.84
Education (Years)	NA	12.41 (2.40)	14.70 (2.19)	t(220) = 7.41***
Marital Status (% Married)	NA	9	41	X^2^ (1, N = 222) = 31.10***
Living Status (% Residing Alone/Residing with Someone Else/Residing in Board and Care)	NA	16/35/48	21/79/0	X^2^ (1, N = 222) = 70.71***
Social Position (HISP) ^b^	1–5	4.71 (.73)	3.47 (1.06)	z = -9.21***
Highest Position (HISP) ^b^	1–5	3.75 (.74)	2.82 (.73)	z = -8.38***
Longest Position (HISP) ^b^	1–5	3.80 (.76)	2.97 (.81)	z = -7.20***
Personal Income^a^	0–8	1.61 (.94)	3.69 (1.74)	z = -8.24***
Family Income^a^	0–8	2.17 (1.48)	4.82 (2.09)	z = -6.45***
Antipsychotic Medication (DDD)	NA	1.81 (1.55)	NA	NA
Ethnicity (% non-Latino Caucasian)	NA	50.00%	60.38%	X^2^ (4, N = 222) = 4.37
**Severity of psychopathology, cognition, and well-being**				
Positive Symptoms (SAPS Total) ^a^	0–20	5.11 (4.42)	NA	NA
Negative Symptoms (SANS Total) ^a^	0–25	5.90 (5.18)	NA	NA
Depression (CDSS Total) ^a^	0–27	2.69 (3.47)	0.80 (1.47)	t(218) = 5.17***
Anxiety (BSIAS Total) ^a^	0–24	7.56 (6.60)	1.23 (1.86)	t(197) = 8.86***
Physical Well-Being (SF-36) ^a^	20.1–57.9	43.55 (10.82)	52.11 (8.88)	t(194) = 6.08***
Mental Well-Being (SF-36) ^a^	17.3–62.1	43.94 (11.22)	53.65 (7.38)	t(194) = 7.24***
Executive Functioning^a^	NA	-0.32 (0.72)	0.54 (0.58)	t(220) = 9.91***
Loneliness (UCLA-3) ^a^	20–80	46.12 (10.98)	34.65 (10.41)	t(220) = 7.97***
**Positive psychological characteristics**				
Happiness (CES-D Happiness Total) ^a^	0–12	7.48 (3.14)	10.02 (2.41)	t(196) = 6.43***
Resilience (CDR Total) ^a^	0–40	24.60 (8.10)	32.50 (6.24)	t(192) = 7.53***
Optimism (LOT-R Total) ^a^	6–30	20.36 (3.99)	23.80 (4.50)	t(195) = 5.69***
Perceived Stress (PSS Total) ^a^	0–40	18.89 (6.51)	11.29 (5.46)	t(193) = 8.74***
Satisfaction with Life (SWLS Total) ^a^	5–35	20.02 (7.44)	23.21 (7.39)	t(195) = 3.01**

*Note*.

** < .01;

*** < .001;

HISP = Hollingshead Index of Social Position; DDD = Defined Daily Dose; SAPS = Scale for the Assessment of Positive Symptoms; SANS = Scale for the Assessment of Negative Symptoms; CDSS = Calgary Depression Scale for Schizophrenia; BSIAS = Brief Symptom Inventory, Anxiety Scale; SF-36 = 36-item Short Form Health Survey; UCLA-3 = University of California, Los Angeles Loneliness Scale, Third Version; CES-D Happiness = Center for Epidemiological Studies—Depression; CDR = Connor-Davidson Resilience; LOT-R = Life Orientation Test—Revised; PSS = Perceived Stress Scale; SWLS = Satisfaction with Life Scale;. Personal and family income were coded according to the following income levels: (1) < $10,000; (2) $10,000–$19,999; (3) $20,000–$34,999; (4) $35,000–$49,999; (5) $50,000–$74,999; (6) $75,000–$99,999; (7) $100,000–$149,000; and (8) ≥ $150,000; Social position was classified as follows: (1) upper, (2) upper-middle, (3) middle, (4) middle-lower, and (5) lower socioeconomic status;

^a^ Higher values indicate higher levels of the measured construct;

^b^ Higher values indicate lower levels of the measured construct

### Factor analyses

The chi-square index of model fit was significant for all factor models other than the bifactor model in both NC and schizophrenia groups (Table 3). Notably, the Chi-square goodness-of-fit index is often significant among adequately fitting models due to its sensitivity to large sample size [29]. Therefore, additional fit measures were inspected to determine model fit and comparative superiority. In both NC and schizophrenia groups, the three-factor model exhibited near acceptable fit while the bifactor model exhibited consistently acceptable fit according to Hu and Bentler [66] criteria. Of these two models, the bifactor model exhibited superior relative fit across all indices. Therefore, the bifactor model was deemed the best-fitting model in both groups.

**Table 3:** Goodness-of-fit indices for factor models of the UCLA Loneliness Scale in schizophrenia and non-psychiatric comparison (NC) groups.

Model	X^2^	df	CFI	TLI	RMSEA (90% CI)	SRMR
**Schizophrenia (n = 116)**						
One Factor	289.83***	170	.759	.731	.078 (.063-.093)	.138
Two Factor	270.54***	169	.796	.771	.072 (.056-.088)	.097
Three Factor	198.75*	167	.936	.927	.041 (.005-.061)	.072
Bifactor	169.89	150	.960	.949	.034 (.000-.057)	.053
**Non-Psychiatric (n = 106)**						
One Factor	264.11***	170	.826	.806	.073 (.055-.089)	.076
Two Factor	237.42***	169	.874	.858	.062 (.042-.080)	.069
Three Factor	206.017*	167	.928	.918	.047 (.019-.067)	.062
Bifactor	178.24	150	.948	.934	.042 (.000-.065)	.050

*Note*.

* < .05;

*** < .001;

CFI = comparative fit index; TLI = Tucker-Lewis; RMSEA = root mean square error of approximation; SRMR = standardized root mean residual

Given that factor analyses revealed a single underlying loneliness dimension after accounting for method effects, Cronbach’s alpha was calculated on the total score of the UCLA-3 as a measure of internal consistency. This total score exhibited an alpha coefficient of .94 (95% CI = .92–.95) in the NC group and .90 (95% CI = .88–.93) in the schizophrenia group. These values represent an excellent level of internal consistency [79].

### Invariance testing across groups

**Fig 1:**
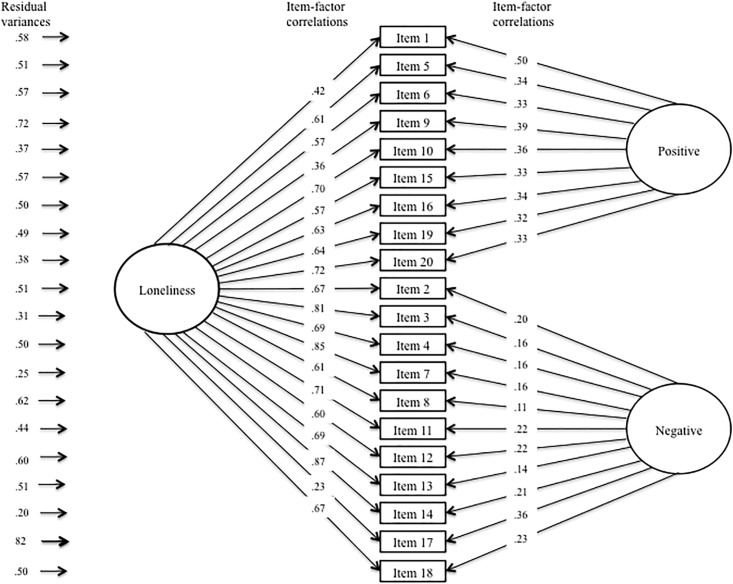
Completely standardized factor model of the UCLA-3 in the non-psychiatric comparison (NC) group. *Note*. Depicted are the completely standardized parameter estimates of the final measurement invariance model; equality constraints were imposed on the unstandardized parameter estimates.

**Fig 2:**
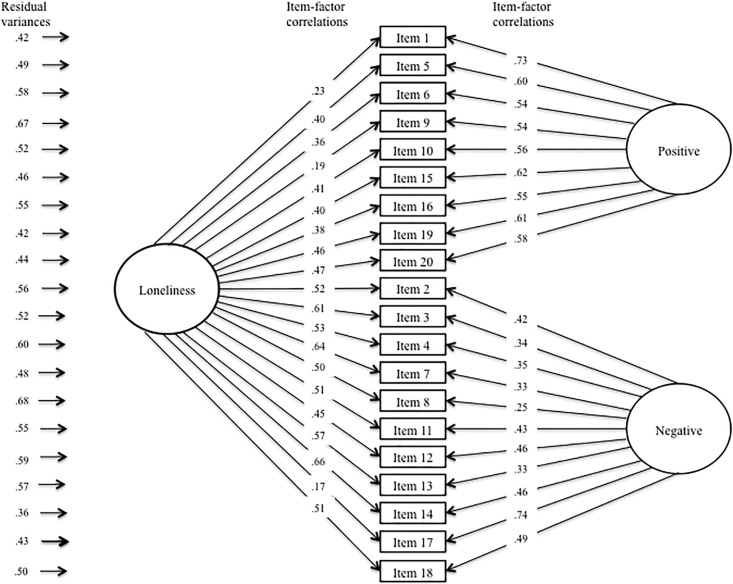
Completely standardized factor model of the UCLA-3 in the schizophrenia group. *Note*. Depicted are the completely standardized parameter estimates of the final measurement invariance model; equality constraints were imposed on the unstandardized parameter estimates.

Table 4 presents fit information for invariance assessment of the UCLA-3 across NC and schizophrenia groups. Configural invariance was established by determining that the same factor model—i.e., Russell’s [30] bifactor model—exhibited acceptable fit for both groups separately and after combining the two groups into a single, larger sample. Inspection of Table 3 reveals that the change in CFI values did not exceed .01 in successive models in the weak or strong invariant models. Taken together, this suggests that the UCLA-3 exhibited the same factor structure (configural invariance), level of item-factor loadings (weak invariance), and item intercept levels (strong invariance) across groups, thus establishing measurement invariance of the UCLA-3. Figs 1 and 2 present depictions of the completely standardized final factor model of the UCLA-3 after imposing measurement invariance across groups.

**Table 4:** Fit statistics for invariance assessment.

Model	X^2^	df	*p*	Δ X^2^	Δ df	*p*	RMSEA (90% CI)	CFI	ΔCFI	TLI	ΔTLI	Pass?
Measurement Model Estimates												
Non-psychiatric	178.24	150		–	–	–	.042 (.000-.065)	.948	–	.934	–	–
Schizophrenia	169.89	150		–	–	–	.034 (.000-.057)	.960	–	.949	–	–
Configural Invariance	343.84	297	.032	–	–	–	.038 (.012-.054)	.955	–	.942	–	Yes
Weak Invariance	385.63	337	.035	–	–	–	.036 (.011-.052)	.953	.002	.947	.005	Yes
Strong Invariance	403.26	354	.036	–	–	–	.036 (.010-.051)	.952	.001	.949	.002	Yes
Latent Model Estimates												
Latent Variance	405.017	357	.040	1.757	3	.624	.035 (.008-.051)	.953	.001	.950	.001	Yes
Latent Means	597.823	360	< .001	192.806	3	< .001	.077 (.066-.088)	.769	.184	.757	.193	No

CFI = comparative fit index; TLI = Tucker-Lewis; RMSEA = root mean square error of approximation; df = degrees of freedom

Subsequent models tested the equivalence of latent parameter estimates—i.e., latent factor variances and means—across groups. While the equivalent latent variance model did not exhibit a significant change in X^2^, the equivalent latent means model did, indicating group differences on the latent factor means underlying the UCLA-3. As can be seen in Table 3, the mean levels of the Loneliness, Positive Wording, and Negative Wording latent factors all differed across groups. After controlling for item wording-related method effects, the schizophrenia group reported a .954 standard deviation higher mean level of loneliness than the NC group.

### Association of loneliness with sociodemographics and clinical characteristics and positive psychological characteristics

The overall pattern of correlations between the UCLA-3 total score and demographic, clinical, and positive psychological variables was similar in NC and schizophrenia groups (Table 5)—i.e., in both NC and schizophrenia groups, loneliness correlated positively with current social position, depression, anxiety, and perceived stress, and negatively with mental well-being, happiness, resilience, optimism, and satisfaction with life. In contrast, age and personal income correlated negatively and longest held social position correlated positively with loneliness in the NC, but not in the schizophrenia group. However, among individuals with schizophrenia, younger age of schizophrenia onset and positive, but not negative, symptom severity were associated with loneliness. Comparisons of the magnitude of correlation coefficients in the NC versus schizophrenia groups revealed significantly higher magnitude correlations among the NC versus schizophrenia groups in terms of the correlations between loneliness and age, optimism, and satisfaction with life. Magnitude of correlation coefficients across groups did not differ on any other sociodemographic factor other than age. In addition, loneliness did not differ across individuals who resided alone (M = 37.18, SD = 10.69) or resided with someone else (M = 33.99, SD = 10.30) in the NC group, *t*(104) = 1.29, *p* = .20, or who resided alone (M = 47.95, SD = 10.14), resided with someone else (M = 47.44, SD = 13.54), or resided in a Board and Care facility (M = 44.54, SD = 8.96) in the schizophrenia group, *F*(2,113) = 1.14, *p* = .32.

**Table 5:** Correlations between the UCLA-3 total score and important demographic, clinical, and positive psychological variables.

Measure	Correlation		
	Schizophrenia (95% Confidence Interval)	Non-Psychiatric (95% Confidence Interval)	Wilcox-Muska test
**Sociodemographic and clinical characteristics**			
Age	-.07	.24*	-.56—-.05*
Age at Illness Onset	-.19*	–	–
Gender	-.03	-.01	–
Education (Years)	-.09	-.08	-.29—.22
Marital Status	-.01	-.08	-.22—.34
Social Position (HISP)	.19* (0.0—.39)	.23* (.07—.39)	–
Highest Position (HISP	.14 (-.04—.33)	.18 (-.02—.34)	–
Longest Position (HISP)	.12 (-.08—.29)	.20* (.04—.37)	–
Personal Income	-.01 (-.21—.18)	-.33** (-.5—-.16)	–
Family Income	-.25* (-.5—-.1)	-.18 (-.38—.03)	–
Antipsychotic Medication (DDD)	.06	–	–
**Severity of psychopathology, cognition, and well-being**			
Positive Symptoms (SAPS Total)	.32***	–	–
Negative Symptoms (SANS Total)	.06	–	–
Depression (CDSS Total)	.46***	.42***	-.22—.30
Anxiety (BSIAS Total)	.39***	.36**	-.25—.34
Executive Function Composite	.11	-.10	-.06—.46
Physical Well-Being (SF-36)	-.13	-.31**	-.18—.47
Mental Well-Being (SF-36)	-.40***	-.45***	-.21—.32
**Positive psychological characteristics**			
Happiness (CES-D Happiness Total)	-.41***	-.61***	-.03—.39
Resilience (CDR Total)	-.36**	-.54***	-.06—.41
Optimism (LOT-R Total)	-.31**	-.57***	.02—.52*
Perceived Stress (PSS Total)	.42***	.57***	-.40—.11
Satisfaction with Life (SWLS Total)	-.24*	-.61***	.12—.62*

*Note*.

* < .05;

** < .01;

*** < .001;

DDD = Defined Daily Dose; SAPS = Scale for the Assessment of Positive Symptoms; SANS = Scale for the Assessment of Negative Symptoms; CDSS = Calgary Depression Scale for Schizophrenia; BSIAS = Brief Symptom Inventory, Anxiety Scale; SF-36 = 36-item Short Form Health Survey; CES-D Happiness = Center for Epidemiological Studies—Depression; CDR = Connor-Davidson Resilience; LOT-R = Life Orientation Test—Revised; PSS = Perceived Stress Scale; SWLS = Satisfaction with Life Scale

## Discussion

To our knowledge, this was the first study to explore the factor structure and psychometric properties of the UCLA-3 among individuals with schizophrenia, as well as being the first to evaluate the equivalency of this measure across NC and schizophrenia groups. Comparisons between previously proposed factor models of this scale revealed that a bifactor model in which all items loaded on a general loneliness factor and one of two orthogonal method factors exhibited the best fit within both schizophrenia and NC groups. Furthermore, the UCLA-3 exhibited equivalent measurement of these latent factors across groups and excellent internal consistency within both groups. Taken together, these results indicate that, after accounting for method effects related to item wording, loneliness in schizophrenia and NC individuals is a unidimensional construct, that this construct is similar across groups, and that the UCLA-3 measures this construct in an equivalent fashion across groups. This indicates that the UCLA-3 is appropriate for making cross-group comparisons between NC and schizophrenia groups.

These factor analytic findings supporting a bifactor model are consistent with several previous studies of the UCLA-3 conducted in NC samples [30, 37]. Others, however, have found one [32], two [33, 80], and three [31, 34, 35] factor models as best fitting this measure in NC samples. However, many of these conflicting studies were conducted on prior versions of the UCLA Loneliness Scale [32, 33, 35, 80], which used more complex wording (e.g., double negatives) and so, may not generalize to the UCLA-3. In addition, none of these alternative models have previously been compared to Russell’s bifactor model. Comparison to this latter model was particularly important because apparent multidimensionality can often arise in scales due to failure to control for method effects or item content groupings [36]. The findings of the current study suggest that, after accounting for method effects due to item wording, intrapersonal, interpersonal, and broader group-level feelings of loneliness are strongly inter-related and cannot be disentangled among persons with schizophrenia and NC individuals.

The similarity in the construct of loneliness across NC and schizophrenia groups was further supported by the similarity in their profiles of loneliness correlates. Specifically, loneliness was similarly positively correlated with current social position, depression, anxiety, and perceived stress, and negatively correlated with mental well-being, happiness, and resilience in both groups. Moreover, the magnitude of correlation coefficients across groups did not differ for gender, education, marital status, highest and longest social status position, personal and family income, executive functioning, or physical well-being. This similar profile of correlates occurred despite differences in mean levels of loneliness across groups, with individuals with schizophrenia reporting mean levels of loneliness nearly a standard deviation higher than those in NC persons. These differences in mean levels are consistent with those seen in several prior studies [21–25]. Overall, these findings suggest that while loneliness is more severe in schizophrenia, it likely operates in a broadly similar fashion among NCs and individuals with schizophrenia. In particular, loneliness appears to be accompanied by a similar increase in perceived stress, presumably due to enhanced vigilance for social threat, and is associated with worse emotional health in both groups. This further suggests that previously proposed cognitive biases accompanying loneliness in NC populations might also be present in schizophrenia. Loneliness in schizophrenia is thus likely not a mere proxy for some other schizophrenia-related deficit (e.g., depression, negative symptoms, etc.), but rather is a distinct phenomenon with clinically meaningful negative effects. Given the high prevalence and negative impact of loneliness in schizophrenia, additional clinical attention to this important phenomenon is warranted.

Despite this overall similarity, there were a few differences in the magnitude of correlations across groups. Specifically, NC individuals exhibited a stronger positive association between loneliness and age than individuals with schizophrenia, for whom this correlation was not significant. A likely explanation for this finding is that the age-related socio-environmental factors that contribute to the association of age with loneliness in the general population may be less relevant in schizophrenia, such as marital quality and losing a partner [81]. In addition, NCs exhibited a stronger negative association between loneliness and optimism and satisfaction with life than persons with schizophrenia, despite the statistical significance of both of these correlations within the two groups. It may be the case that, due to the larger number of clinical (e.g., positive symptoms, negative symptoms, depression) and socio-environmental (e.g., unemployment, financial concerns) challenges in schizophrenia, there are more determinants of optimism and satisfaction with life, leading any single determinant to have a relatively weaker impact in schizophrenia as opposed to the general population.

More broadly, this latter point speaks to the potential for the unique clinical features of schizophrenia to alter the correlation of loneliness with other sociodemographic, clinical, and positive psychological factors. For instance, positive symptoms, which are positively correlated with loneliness, often diminish over the course of the lifespan among individuals with schizophrenia [82]. It is thus possible that diminished levels of positive symptoms in older age may in turn reduce the correlation of loneliness with age. We re-ran the current analyses statistically adjusting for unique clinical features of schizophrenia that were significantly correlated with loneliness. Positive symptoms were the only features that fit these criteria. Statistically adjusting models for positive symptoms did not substantively change the correlations within the schizophrenia group or the significance of differences in magnitude of correlation coefficients across schizophrenia and NC groups, indicating that this unique clinical feature does not confound the results of the current study. Nonetheless, there are other clinical features that are unique to schizophrenia, such as internalized stigma, that we did not collect data on and thus were not able to statistically control for. Future research aimed at evaluating loneliness across schizophrenia and non-schizophrenia groups should attempt to ascertain the impact of and, when necessary, control for these unique factors.

In addition, it should be noted that schizophrenia and NC groups differ not only in diagnostic status, but also in the social environments that they inhabit. In this regard, it may be necessary to distinguish between the direct effects of schizophrenia on loneliness versus the effects of schizophrenia on socioeconomic status, as the two paths may have different implications for treatment or policy. In general, individuals with schizophrenia are less likely to be employed, married, and live with someone else and typically have lower social status and personal incomes [8–10]. In addition, the communities in which they reside tend to have fewer opportunities and resources [11–13]. Taken together, it is likely that individuals with schizophrenia are objectively more socially isolated than NC individuals, which may impact their subjective perception of social isolation (i.e., loneliness). While research in the general population has typically not found that objective social isolation and loneliness are correlated with one another (largely due to individual differences in the need and desire for social contact), this issue has not been explored in schizophrenia [83]. In the current study, we found that most aspects of one’s social environment were not correlated with loneliness in the schizophrenia group, including marital status, living situation, and personal income, although current social status position and family income were. In addition, the magnitude of correlation coefficients of loneliness with each social factor examined in the current study did not differ across schizophrenia and NC groups, suggesting that these social factors have a similar impact in both populations.

Notably, however, one’s social environment consists of many more relevant characteristics than those measured in the present study. These characteristics include the physical aspects of one’s community (e.g., availability of community resources), social aspects of one’s community (e.g., community cohesion, stability/turnover rates of residence, quantity and quality of social relationships), and psychological aspects of one’s community (e.g., sense of community belonging), among others [84]. Previous research has found that relative to community-matched non-psychiatric individuals, individuals with schizophrenia and other psychiatric disabilities often report lower use of and engagement with community resources [11, 12] and lower quality and quantity of social relationships [11, 12, 85], but report a similar sense of community belonging [13, 85] and satisfaction with life [13]. These findings parallel the lack of an association between objective and subjective aspects of social isolation found in the general population. However, the similarity in subjective perception of community belonging across community-matched schizophrenia and non-psychiatric groups is somewhat inconsistent with the finding of higher levels of loneliness among individuals with schizophrenia relative to non-psychiatric individuals, which was found in the current study and in several previous studies [21–25]. It may be the case that subjective sense of belonging within the community is related to but only partially overlaps with the construct of loneliness. Consistent with this, some factor models of loneliness distinguish between “Isolation” (feelings of aloneness, rejection, and withdrawal), “Relational Connectedness” (feelings of familiarity, closeness and support), and “Collective Connectedness” (feelings of group identification and cohesion), with only this latter dimension likely overlapping with sense of community belonging [34]. Future research should explore the role of these finer-grained physical, social, and psychological aspects of the social environment in relation to loneliness in schizophrenia.

Many of the correlations with loneliness in NC and schizophrenia groups found in the current study are consistent with those found in previous research. Previous research has similarly shown an association of loneliness with poorer emotional and physical health, perceived stress, and reduced quality of life, as well as mild increases in loneliness in middle adulthood in the general population [3, 4, 86]. In contrast, inconsistent with previous research, we did not find an association between loneliness and cognition, which may be due to the relatively low levels of loneliness within our NC group. Within the schizophrenia group, similar to previous research, loneliness was positively associated with depression [25], anxiety [28], and positive symptoms [26, 28], and negatively correlated with satisfaction with life [16, 17]. We expanded on this research by demonstrating that loneliness was associated with a broader range of clinical and positive psychological characteristics, including age of schizophrenia onset, mental well-being, perceived stress, optimism, resilience, and happiness.

One limitation of the present study is that the sample size was somewhat small for factor-analytic purposes [29]. Nonetheless, we were able to derive an adequately fitting factor model and document significant correlates of loneliness. A second limitation is that we were unable to explore all psychometric features of the UCLA-3; additional research is needed to evaluate the test-retest reliability, convergent validity, and discriminant validity of this measure among people with schizophrenia. Thirdly, our results may not generalize to patients with more severe illness such as the institutionalized ones or to people from other racial/ethnic groups. Also, we do not know if our findings are specific to schizophrenia or also apply to other serious mental illnesses. Finally, we did not collect information on physical, social, and psychological aspects of the community integration or neighborhood characteristics. These factors may impact levels of loneliness, and thus should be collected as part of future studies.

Research among NC samples has shown that loneliness can lead to negative physical, cognitive, and emotional health outcomes over time [4]. Although cross-sectional results show that loneliness is associated with poor physical and emotional health in schizophrenia, it remains to be seen if loneliness predicts declines in these areas longitudinally [15, 23, 25, 26]. Prospective longitudinal research may also help clarify the factors that may predispose individuals with schizophrenia to loneliness, including psychological factors (e.g., distorted beliefs) and/or social-environmental factors (e.g., poverty, community integration, and housing circumstances). Finally, given its high prevalence and association with factors contributing to worse quality of life and physical and emotional health in schizophrenia, loneliness may be a promising target for interventional research.

Despite the above limitations, the present findings are important in documenting similarities in the construct of loneliness and its correlates across NC and schizophrenia groups. In both groups, loneliness was associated with increased perceived stress and worse emotional health and satisfaction with life. Loneliness thus appears to be distinct from other schizophrenia-related deficits and is an important yet under-recognized problem in schizophrenia. Loneliness needs to be studied as a specific target for intervention research in persons with this serious mental illness.

## Supporting information

S1 File De-identified data set. (XLSX)
